# 肺癌“一体化诊疗、全程管理”模式的发展现状与思考——基于四川省肿瘤医院肺癌MDT团队经验

**DOI:** 10.3779/j.issn.1009-3419.2020.101.12

**Published:** 2020-04-20

**Authors:** 润 向, 强 李

**Affiliations:** 610041 成都，电子科技大学医学院附属肿瘤医院，四川省 癌症防治中心，四川省肿瘤医院·研究所，胸外中心 Department of Thoracic Surgery, Sichuan Cancer Hospital & Institute, Sichuan Cancer Center, School of Medicine, University of Electronic Science and Technology of China, Chengdu 610041, China

**Keywords:** 肺肿瘤, 多学科综合诊疗协作组, 全程管理, Lung neoplasms, Multi-disciplinary treatment, Full-course management

## Abstract

随着医疗水平的提高，疾病诊疗模式也发生了改变。肺癌是最常见的恶性肿瘤，也是诊疗极其复杂的疾病之一。多学科综合诊疗（multi-disciplinary treatment, MDT）协作组在肿瘤的诊疗中具有独特优势，但在国内并未得到广泛应用，故结合我院MDT团队诊疗和管理经验进行讨论及综述，阐述肺癌“一体化诊疗、全程管理”模式的发展现状及推广价值。

## Abstract (en)

With the improvement of medical standards, the pattern of disease diagnosis and treatment has also changed. Lung cancer is the most common malignancy and one of the most complicated diseases to diagnose and treat. Multi-disciplinary treatment (MDT) group has unique advantages in the diagnosis and treatment of tumors, but it has not been widely used in China. Therefore, it is discussed and summarized based on the diagnosis and treatment and management experience of our MDT team. The development status and promotion value of the "integrated diagnosis and treatment, full-course management" model of lung cancer are described.

肺癌是发病率和死亡率增长最快、对人群健康和生命威胁最大的恶性肿瘤之一。随着精准疗法和免疫治疗时代的到来，肺癌的生存期也相应得到延长，但目前发达国家肺癌的5年生存率也仅为14.7%-21.7%^[1, 2]^，其中，转移可能是导致肺癌治疗失败和患者死亡的首要原因^[3, 4]^。肺癌是一种全身性疾病，肺癌的治疗不仅包括原发灶的治疗，还包括转移灶、合并症的治疗，同时针对药物副作用、患者心理的管理也不容忽视。可见，肺癌诊治涉及到多个学科，各阶段的治疗方案截然不同，而患者管理也是一个漫长的过程；如何整合各个学科优势、共同参与患者的管理，将是临床医生、医院管理专家亟待解决的问题和重要探索方向。

## 1 多学科团队组建

肿瘤患者的诊疗涉及学科广、全程管理时间跨度大，组建多学科团队对肿瘤患者进行诊疗与管理，将是未来治疗癌症的发展方向。肿瘤患者的诊疗从诊断肿瘤开始，到最后的临终关怀，涉及到影像学、病理学、外科、放疗科、化疗科、检验科、心理学、营养学、缓和医学等多方面内容，不是单一学科能够承担的。随着癌症治疗水平的提高，患者的生存期不断延长，对提高生命质量的要求不断增加，除了抗肿瘤治疗外，还需要医师对患者进行疼痛及其他症状的处理，并考虑患者的生理、心理和精神方面的健康。可见，癌症患者的管理，不仅涉及到患者，还关系到其家庭成员；管理的不仅是患者的生存期，还包括生活质量、营养状态以及患者心理等方方面面。因此，诊疗一体化有利于多学科共同解决肿瘤诊疗问题，全程化管理也是提高肿瘤患者生存率和生存质量的保障。

### 1.1 一体化诊疗团队

以肺癌为例，其多学科综合诊疗（multi-disciplinary treatment, MDT）团队至少包括影像科、病理科、胸外科、化疗科、放疗科等相关科室。接诊肺癌患者后提交多学科团队讨论，首先由影像科给出临床诊断，胸外科医生判断手术价值及方案；若涉及到疑难病例或基因改变的情况需要病理科医生的参与；手术后的治疗方案也应由多学科团队制定。随着人口老龄化进程加快，我们面临的高龄以及患有合并症的肺癌患者日益增多，治疗过程中可能会伴随着心血管系统、内分泌系统以及呼吸系统等并发症。因此，肺癌MDT团队需要实时扩大参与的学科，包括心血管、呼吸、内分泌等专业医师的参与，以保证患者得到全面的诊治。

### 1.2 全程化管理团队

肺癌患者的全程管理从诊断开始，涉及到治疗、随访复诊的全程，直至最后的临终关怀，同样需要多学科团队的参与。肿瘤患者在诊断、治疗以及随诊过程中心理变化大，实时地进行心理学干预可能改善治疗效果；肿瘤治疗中和治疗后，要进行随访和护理，如何保证患者的营养水平也是一大问题，因此营养师、护理团队的加入是必要的。肺癌治疗过程中，转移是导致治疗失败和患者死亡的首要原因，其中，骨是肺癌最常见的血性转移部位之一，约10%-50%的肺癌患者治疗过程中会发生骨转移，骨转移常预示患者生活质量的下降和生存期的缩短^[5-7]^。尤其是骨转移引起的骨痛、病理性骨折、脊髓压迫、高钙血症及相关治疗带来的痛苦等，严重影响患者的生活质量。因此，加入肿瘤疼痛管理，缓和医学等专业参与患者的治疗，同时对高龄及合并症患者加入心血管、呼吸等专业医师的参与，以保证患者得到全面的诊治，获得最佳的生活质量。

## 2 一体化诊疗实施方案

### 2.1 肺癌患者“一体化诊疗”模式经验分享

为了提高肺癌疑难病例的诊疗效率和质量，我院于2013年8月率先在西南地区成立肺癌多学科综合诊疗协作组，简称MDT。提供以疑难复杂肺癌患者为中心的MDT服务模式，推进肺癌规范化诊疗，以提升西南地区肺癌诊疗决策质量。我院肺癌“一体化诊疗”模式由医务部协调，以肺癌MDT团队为基础，开展疑难复杂肺癌病例的诊治工作。肺癌MDT团队包括胸外科、肿瘤内科、放疗科、影像科、病理科、检验科、营养科、中医科、护理组、个案管理、综合内科等在内的十余个学科。团队核心成员中，高级职称占100%，硕博士学历者占70%；同时制定了我院《肺部肿瘤MDT入组标准》、《原发性肺癌MDT临床实践共识（2015版）》及《肺癌患者MDT诊疗流程》，门诊患者和住院患者均可申请MDT讨论，团队每周固定时间开展讨论。

### 2.2 基于患者报告结局（patient-reported outcome, PRO）的随访管理模式在MDT中的应用

由于医学模式转变，我们提供的医学服务将逐渐从“以疾病为导向”的主流医学，走向“以生存时间及生活质量为导向”的新型医学转换。对于肿瘤患者，医生不仅需扮演治愈疾病的角色，更应注重对患者的人文关怀；不能单一追求患者无瘤生存时间，应在保障生存质量的前提下延长生存时间，乃至带瘤生存时间^[8]^。因此，以传统“治病”为基础的生存质量评估系统，没有涵盖患者对“康复”的期望，已无法满足现代肿瘤患者治疗全程生活质量评估。研究认为由患者本人评价自身症状功能状态比由医护人员测量更为准确^[9]^。PRO是在未经医疗专业人士解释的情况下，直接来源于患者对自身健康状况和治疗结果的主观评价^[10]^，作为美国食品药品监督管理局（Food and Drug Administration, FDA）建议使用的新型结局指标^[11]^，因其在测量患者日常功能及症状领域具有可靠性、灵敏性和易操作性，目前已应用在欧美临床研究、药物审批和医疗服务质量评估等方面^[12]^。最新的大规模临床试验^[13]^显示在晚期化疗患者中应用PRO症状监测预警不仅可以改善患者的生活质量，而且可以显著延长患者生存。

肺癌的诊疗和随访是长期过程，患者可能先后接受了手术、放化疗、靶向治疗、免疫治疗以及并发症、合并症的治疗，此过程中可能出现各种各样的症状，评估标准也复杂多样。进入MDT讨论的肺癌患者病情复杂，医师在关注患者生存期的同时，也应尽可能提高患者生存质量。PRO随访管理模式不仅涉及了围手术期症状管理，也涵盖了放化疗后并发症的方方面面，基于患者本人评价自身功能状态可更好地反映其生存质量，也为肺癌全程管理提供新思路，应作为肺癌MDT管理模式的拓展方向。

### 2.3 以MDT模式提升建设癌症区域医疗中心能力

随着循证医学理念推广应用以及对肿瘤生物学特性的深入研究，各学科逐步认识到单一治疗在肿瘤领域的局限性，“多学科综合诊疗（MDT）”理念应运而生。现在MDT诊疗模式已被国内外肿瘤学界的多数学者所认同，它同时顺应了国家癌症区域医疗中心建设要求。MDT模式主要承担疑难危重肿瘤患者的诊疗，诊疗过程中由经验丰富的多个学科专家制定方案、提供高水平医疗技术，年轻医生也会参与到诊疗的全程、学到先进技术与理念；同时，疑难危重患者也是重要的科研方向，更是优秀的临床教学案例。作为肿瘤专科，大力推广MDT模式应用于各癌种将是提升区域医疗中心诊疗能力和影响力的重要策略之一。

## 3 肿瘤患者全程管理

肿瘤患者的管理至少包括疾病健康教育、随访复诊、营养状态监测与干预、康复治疗、心理互动等方面。疾病健康教育不仅要针对患者进行疾病科普，个体化治疗方案以及规范化随访复诊等进行讲座，让患者对疾病、全程诊疗有一个清晰的认识，可更好地配合诊疗及全程管理。同时，我们还要对不同患者进行个体化管理，让患者成为真正的现代医学模式的中心。

### 3.1 个案管理模式在MDT中应用

个案管理（case management）是结合医院各专业领域为患者提供系统性照护服务的工作模式，它针对个体患者的疾病特性及个性化的需求，经过评估、个案界定、计划、实施、评价，通过沟通、协调和资源的连结分配，以团队方式为患者提供整合性的全程照护。个案管理在台湾肿瘤领域中的发展与肿瘤多学科合作的照护模式同时开展，肿瘤个案管理模式保证了多学科合作诊疗的实现，个案管理师在该模式中担任了非常重要的协调者角色。

目前国内各大医学中心对个案管理的实践已经在乳腺癌照护中体现出治疗效果，随访成功率、患者生活质量和治疗满意度方面也显现出显著的优势。越来越多的肺癌患者在就诊时，会希望得到更加权威、全面、优质的医疗服务；在最短的时间拿到报告，以最快的速度确定治疗方案，并且在后续的随访中，希望得到一个可及时回馈信息的医疗服务^[14-19]^。肺癌个案管理非常适合患者的需求，但在国内还处于试探阶段。我院肺癌MDT团队已于2018年10月开展肺癌个案管理工作，虽然工作刚起步，但是因为患者在入院、诊疗、出院、随访等阶段得到医务人员的主动管理，所以其不但得到了最佳治疗，而且满意度也明显提升，该管理模式的优势得以凸显。

### 3.2 营养干预

恶性肿瘤是一种慢性消耗性疾病，肿瘤生长过程需要消耗患者的营养，治疗过程中会出现胃肠道反应、骨髓抑制和免疫功能降低等毒副反应，不但会影响患者的营养吸收，也会间接影响治疗效果。可见，恶性肿瘤患者是营养不良及营养风险的高发人群，重者可导致恶病质。营养风险是指现有的或潜在的与营养有关的因素导致患者出现不良结局的风险，在肿瘤患者中可表现为并发症增多、死亡率增加、住院时间延长、生存质量差等。我们医院肺癌MDT团队的营养师会对每个MDT病例进行营养风险评估，在治疗以及康复过程中给患者提供饮食营养指导，定期开展相应的营养科普讲座及咨询，以此提高患者生活质量、改善生存期。

### 3.3 心理干预

心理状态变化会影响到人的自主系统、各级脑组织和内分泌系统等，通过调节体内的各种激素生物因子，进一步影响身心健康。确诊癌症对患者本人，乃至整个家庭来说都是一个严重心理打击；癌症的治疗如手术、化疗或放疗等过程，以及后续康复过程也会带来一定的负性心理反应，因此有效的心理干预可能是提高癌症患者的生存质量、促进康复的重要手段。癌症患者诊治康复过程长，通过MDT形式将患者的心理干预纳入到全程管理中，结合肺癌个案管理模式让患者获得最好的身心康复，可能是多学科团队发展的又一方向。

## 4 基于“互联网+”肺癌诊疗体系建立及应用前景

基于“互联网+”的医联体诊疗体系建设，是深化医改的重要步骤和制度创新，是推进医疗资源下沉、提升基层服务能力、实施分级诊疗和满足群众健康需求的重要举措。各肺癌MDT团队应依托医联体，利用自身优势，组建基于“互联网+”的覆盖本省、引领区域、辐射全国的诊疗体系，助力肺癌诊治水平的提升，让更多患者获益。应用该诊疗体系，针对患者提供个性化的治疗方案，建立随访管理系统与患者管理系统，通过人工智能（artificial intelligence, AI）语音随访、患者全息档案管理、智能拨号和患者教育，帮助患者管理疾病；激励患者自发上传诊疗数据（报告、医嘱、用药记录等），快速得出基于患者报告的检测结果，建立症状与疾病之间的联系。

目前，我院肺癌MDT团队正在组建“互联网+”肺癌诊疗体系，通过打通患者诊前、诊中、诊后全流程闭环管理，解决医生和患者痛点问题，真正实现“以患者为中心”，增强患者疾病管理。同时，形成了以我院肺癌MDT团队为核心，由省内多家地市级医院MDT团队组成的网络MDT会诊平台，以此提升各类医疗资源利用率和健康管理服务水平，推动优质资源有效下沉，切实提升肺癌诊疗水平，达到同质化治疗。

## 5 思考与展望

随着单一治疗在肿瘤领域的局限性日益凸显，MDT诊疗模式已逐渐被肿瘤学界的多数学者认同^[20]^。但是，目前MDT诊疗的多为疑难危重病例，早期病例较少。然而，对高危因素的人群进行筛查，发现早期肿瘤患者并及早进行干预，是提高肿瘤疗效、预防癌症最有效手段之一。那么，肿瘤筛查、早期肿瘤诊疗是否会成为MDT的下一个发展方向？同时，是否可将分子生物学、代谢组学、免疫学、基因组学等多组学整合于肿瘤的MDT诊疗中？均是需要进一步探索的课题。相信在未来十余年，“诊疗一体化、全程管理模式”会给癌症患者的生存带来明显改善，也将成为癌症诊疗的重要途径。
